# Diagnostic and Prognostic Biomarkers for Myocardial Infarction

**DOI:** 10.3389/fcvm.2020.617277

**Published:** 2021-02-03

**Authors:** Yuling Wu, Nana Pan, Yi An, Mengyuan Xu, Lijuan Tan, Lijuan Zhang

**Affiliations:** ^1^Systems Biology & Medicine Center for Complex Diseases, Center for Clinical Research, Affiliated Hospital of Qingdao University, Qingdao, China; ^2^Department of Cardiology, Affiliated Hospital of Qingdao University, Qingdao, China

**Keywords:** serum biomarkers, myocardial infarction, MI diagnosis, MI prognosis, circulating biomarkers

## Abstract

The incidence of myocardial infarction (MI) increases every year worldwide. Better diagnostic and prognostic biomarkers for clinical applications are the consistent pursuit of MI research. In addition to electrocardiogram, echocardiography, coronary angiography, etc., circulating biomarkers are essential for the diagnosis, prognosis, and treatment effect monitoring of MI patients. In this review, we assessed both strength and weakness of MI circulating biomarkers including: (1) originated from damaged myocardial tissues including current golden standard cardiac troponin, (2) released from non-myocardial tissues due to MI-induced systems reactions, and (3) preexisted in blood circulation before the occurrence of MI event. We also summarized newly reported MI biomarkers. We proposed that the biomarkers preexisting in blood circulation before MI incidents should be emphasized in research and development for MI prevention in near future.

## Introduction

**Figure 1:**
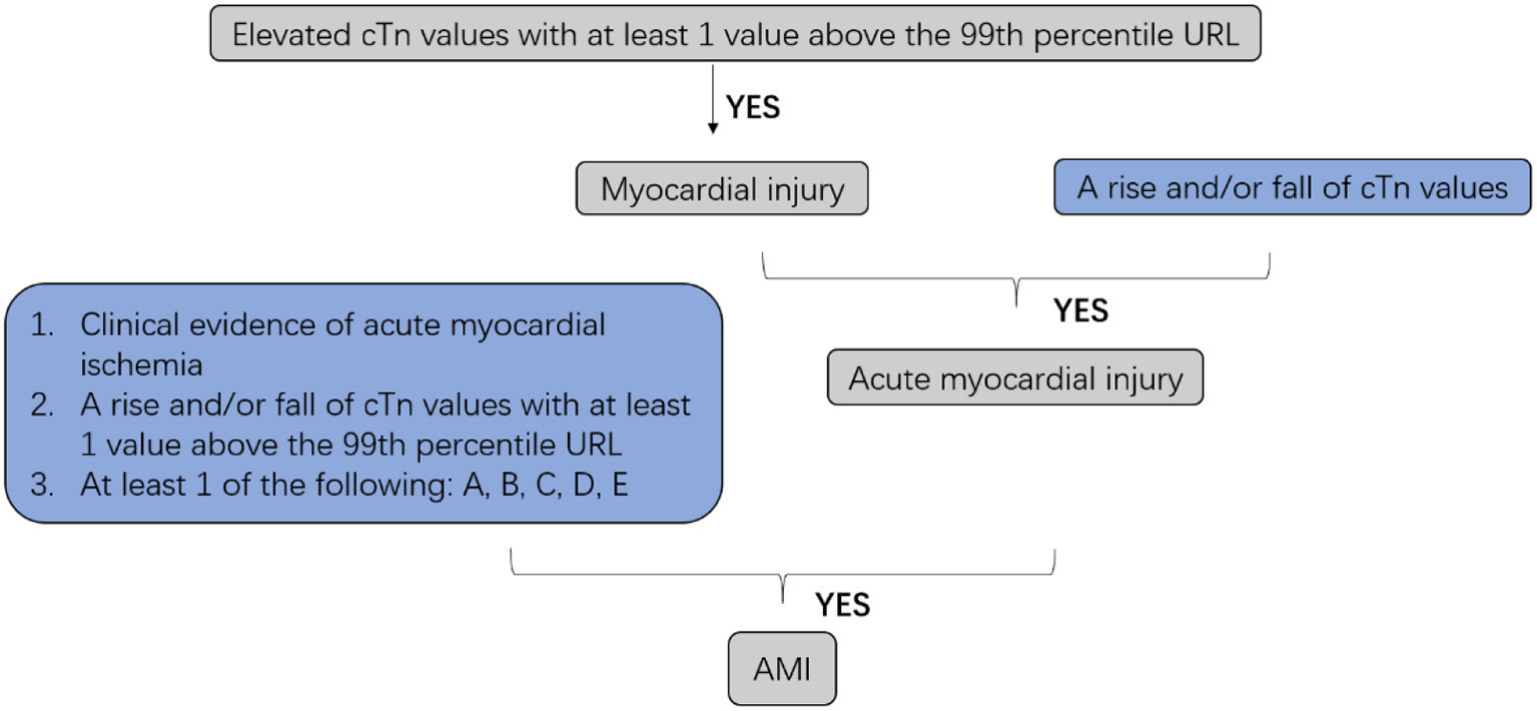
Clinic defined acute myocardial infarction (AMI). cTn, cardiac troponin; URL, upper reference limit; AMI, acute myocardial infarction; A: Symptoms of myocardial ischemia; B: New ischemic ECG changes; C: Development of pathological Q waves; D: Imaging evidence of new loss of viable myocardium or new regional wall motion abnormality in a pattern consistent with an ischemic etiology; E: Identification of a coronary thrombus by angiography or autopsy (not for types 2 or 3 MIs).

**Figure 2:**
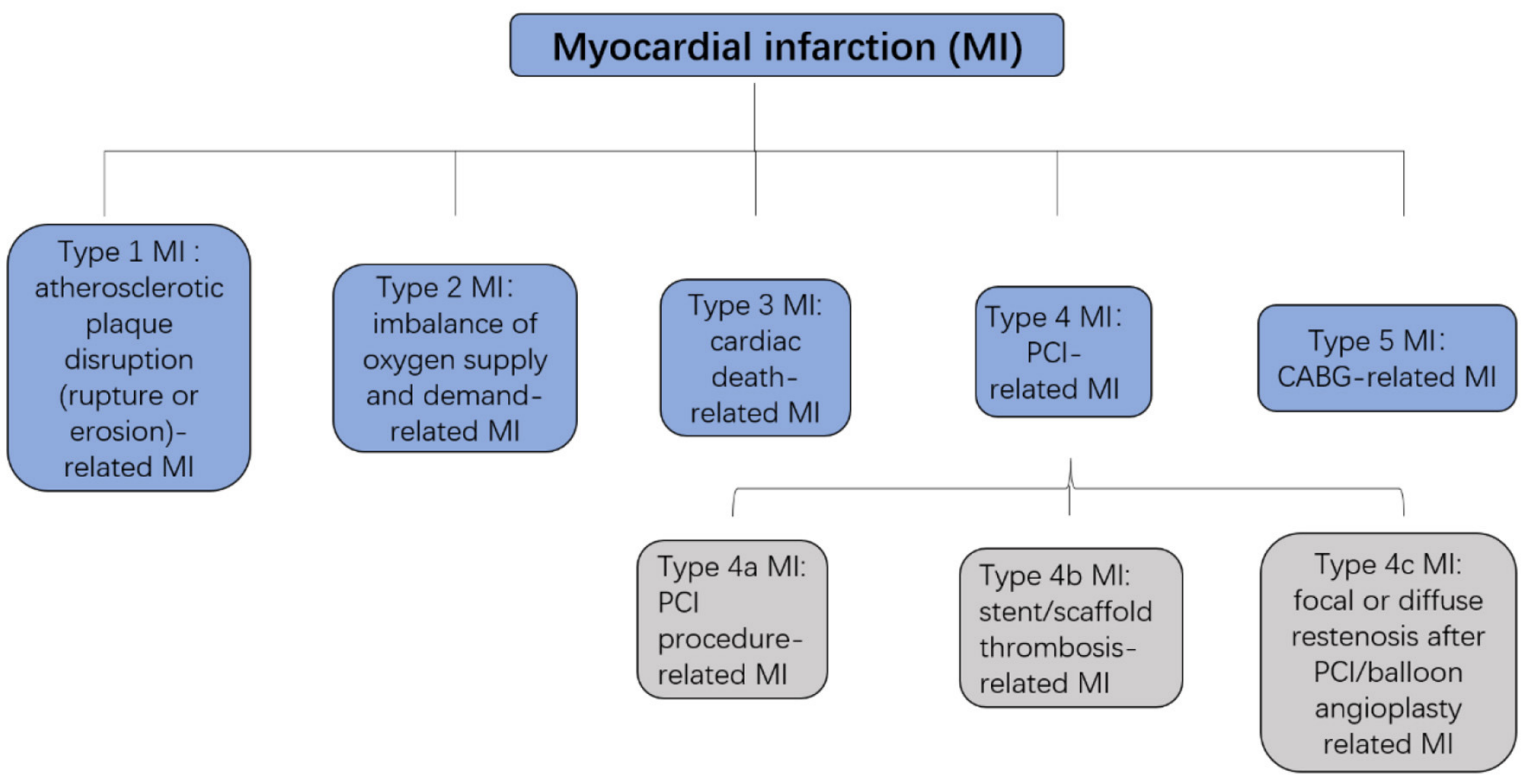
Five types of MI. MI, myocardial infarction; PCI, percutaneous coronary intervention; CABG, coronary artery bypass grafting.

Cardiovascular diseases are a leading cause of mortality in humans, and nearly 20 million individuals worldwide die from acute cardiovascular events every year. Myocardial infarction (MI), also known as a heart attack, is a myocardial injury caused by myocardial ischemia (1). In 2018, the fourth Universal Definition of Myocardial Infarction emphasized the difference between acute myocardial infarction (AMI) and myocardial injury and divided MI into five types (2, 3) (Figures 1, 2).

**Figure 3:**
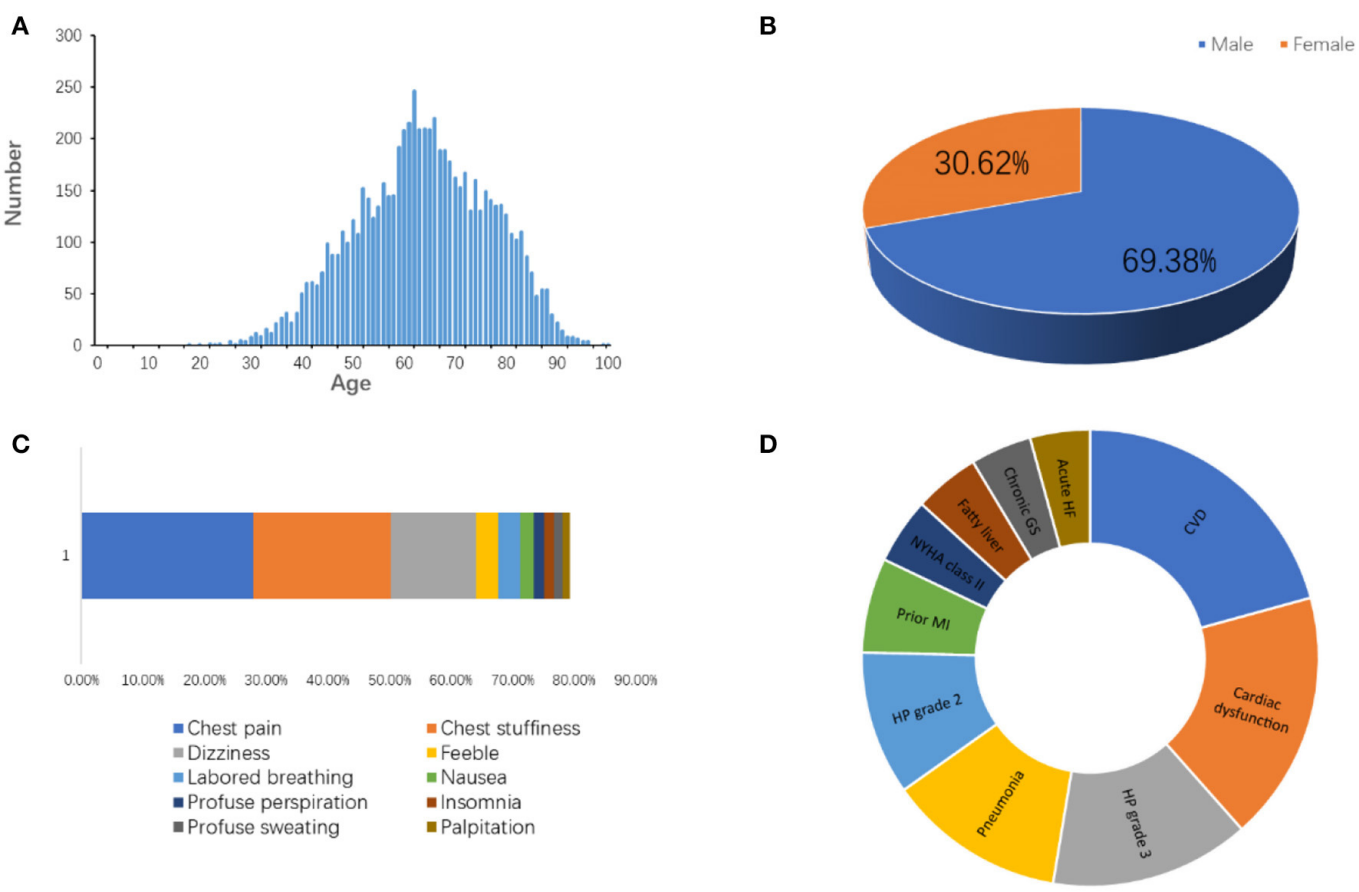
The information of patients diagnosed as MI admitted to the Affiliated Hospital of Qingdao University from 2015 to 2020. **(A)** Age distribution. **(B)** Gender distribution. **(C)** Symptoms. **(D)** Companion diagnosis. CVD, cardiovascular disease; HP, high blood pressure; MI, myocardial infarction; GS, gastritis; HF, heart failure.

Approximately 1.5 million individuals in the United States suffer from MI every year (4). We have followed the information of MI patients admitted to the Affiliated Hospital of Qingdao University in China from January 2015 to August 2020. The total incidents, patient age, gender, symptoms, and companion diagnosis are summarized in Figure 3, which are consistent with published knowledge that the MI patients are largely male ranging from 60 to 75 years old with typical symptoms, such as chest pain, chest stuffiness, and dizziness.

The high incidence of MI results in financial burdens to both families and society and affects the life quality of MI patients.

In addition to ECG, echocardiography, coronary angiography, etc., circulating biomarkers are essential for MI diagnosis. The MI circulating biomarkers have gone through a long process from discoveries to clinical applications. Table 1 summarizes the milestone of the MI biomarker research and development during the past 70 years.

**Table 1:** The milestones of biomarker discoveries for myocardial infarction.

**Years**	**Milestone**	**References**
1954	AST released from the necrotic cardiac myocytes to the circulation could be helpful in diagnosing AMI	(5)
1954–1955	LDH was considered as a useful marker in the diagnosis of AMI	(6, 7)
1978	CK activity was considered a better predictor of myocardial injury and an independent indicator in the diagnosis of AMI for 20 years	(8)
1982	Myoglobin occurs in the first 30 min while it has less specificity	(9)
1982	S100 gradually comes into spotlight and is related to MI	(10–14)
1992	Subtypes of IL play crucial roles in diagnosis and prognosis of MI	(15–21)
1993	“Decreased immunoreactivity for H-FABP may be a good histological marker of damaged cardiomyocytes”	(22)
1997	IGF may protect cardiac function after AMI	(23)
1998	cMyC is expressed in the heart specially	(24–27)
2001	uACR is associated with increased long-term risk of cardiovascular and total mortality in survivors of MI	(28)
2003	Atherosclerosis is proved to be associated with alterations in MMP activity	(29)
2003	MiRNAs are associated to MI	(30–41)
2004	VEGF is associated with diagnosis, infarction size, and clinical outcomes of MI	(42–45)
2007	Hs-cTn is current golden standard for AMI diagnosis	(46)
2013	HPA is associated with acute MI accompanied with elevated white blood cell count	(47)
2013	Copeptin: a new marker in cardiology	(48)
2014	Ischemia-reperfusion injury has been proved to decrease MG53 in heart	(49)
2015	PIK3C2A could affect angiogenesis contributing to the pathophysiology of coronary artery disease	(50)

*AST, aspartate aminotransferase; LDH, lactate dehydrogenase; CK, creatine kinase; IL, interleukin; H-FABP, heart type fatty acid-binding protein; IGF, insulin-like growth factor 1; cMyC, cardiac myosin-binding protein C; VEGF, vascular endothelial growth factor; hs-cTn, high sensitivity cardiac troponin; HPA, heparanase*.

The AMI circulating biomarkers can be divided into three categories: (1) the biomarkers originated from damaged myocardial tissues and released into blood circulation (Table 2); (2) biomarkers with increased levels in blood circulation due to systems reactions after the MI events (Table 3); and (3) biomarkers with abnormal serum levels before the occurrence of MI event (Table 4). Both the strength and weakness of these MI circulating biomarkers will be discussed in the following sections. We also discussed the strengths and weaknesses of newly reported MI biomarkers. We proposed that the biomarkers preexisted in blood circulation before MI incidents should be emphasized in research and development for MI prevention in near future.

**Table 2:** Biomarkers originated from myocardial tissue.

**Abbreviation**	**Full name**	**Characteristics**	**Remarks**	**References**
LDH	Lactate dehydrogenase	^*^Low sensitivity and specificity ^*^Distinguish acute from subacute MI in patients with positive troponins and negative CK or CK-MB	LDH1:LDH2 ratio >1 is specific for AMI	(51)
CK	Creatine kinase	^*^Higher sensitivity and specificity than LDH ^*^Cannot detect minor myocardial injury	^*^MB2:MB1 ≥1.5 is in favor of AMI ^*^CK-MB relative index (CK-MB/total CK^*^100) could be used to diagnose MI ^*^Total CK and CK-MB are related to infarction size and prognosis of MI	(5, 52–54)
–	Myoglobin	^*^Has no specificity so negative values are more meaningful than positive ^*^Rises early after MI	Used to evaluate infarction size and reperfusion	(5, 9, 55)
cTn	Cardiac troponin	Highest sensitivity and specificity among biomarkers applied to clinic	Golden standard	(5, 46)
H-FABP	Heart type fatty acid-binding protein	^*^Sense post-ischemic myocardial reperfusion injury ^*^Prognose relatively long-term post-ischemia	High negative predictive value of H-FABP test can help to rule out AMI earlier	(56–62)
cMyC	Myosin-binding protein C	Rise and fall more rapidly after myocardial injury	To rule in/out AMI more effectively among those presenting early after symptom onset	(27, 63)

**Table 3:** Biomarkers induced by MI incidence.

**Abbreviation**	**Full name**	**Characteristics**	**Remarks**	**References**
ILs	Interleukins	*Targeting IL-1 could be a novel therapy pericarditis associated with inflammasome activation after MI *Related to cardiac remodeling	IL-1Ra may have a predictive effect on MI	(15, 17, 64–66)
IGF-1	Insulin-like growth factor 1	*Reduce adverse cardiac remodeling *Improve ventricular arrhythmia	Cannot be used to diagnose MI	(67–72)
VEGF	Vascular endothelial growth factor	*An independent risk factor for adverse clinical outcomes after AMI *Associate with infarct size in patients with AMI	Different subtypes have various effect	(42, 44, 45)
MMPs	Matrix metalloproteinases	Circulating MMP-28, a predictor for short-term prognosis in patients with MI	–	(73)

**Table 4:** Biomarkers preexisted before MI occurred.

**Abbreviation**	**Full name**	**Characteristics**	**Remarks**	**References**
Glc	Glucose	Significantly increased in AMI	Lack specificity for MI	(4)
AST	Aspartate aminotransferase	Previously used MI biomarker	Lack specificity for MI	(5)
RNAs	RNAs (including microRNAs and LncRNAs)	Involved in every aspects of MI	Next generation biomarker for MI	(30–41)
S100	–	S100 family are essential in diagnosis and prognosis of MI.	–	(74–76)
HPA	Heparanase	Participate in mediating pathological process of MI	A predictive marker for high thrombus burden in patients with STEMI	(47, 77)
PIK3C2A	–	Low expression of PIK3C2A gene is an independent risk factor and could serve as a potential biomarker to predict risk of AMI	–	(78)
Copeptin	–	Combinated with hs-cTnI to detect suspected ACS patients with low hs-cTnI	Gender-specific	(79, 80)
Mitsugumin 53	MG53	Elevated serum MG53 levels shows a significant adverse outcome after a 3-year follow-up among patients with STEMI	–	(81)
The serum albumin-to-creatinine ratio	sACR	An independent prognostic marker and a useful marker for early risk stratification of patients with AMI	–	(82)

## Biomarkers

### Biomarkers Originated From Myocardial Tissues

#### Lactate Dehydrogenase

Lactate dehydrogenase (LDH) was considered as a useful biomarker in diagnosing AMI (51, 83). LDH has five isoenzymes. LDH-1 is expressed in the heart but it is not heart-specific (83). Circulating LDH-1 increases within 6–12 h from onset of chest pain. It peaks at 1–3 days and returns to normal within 8–14 days. Due to its low sensitivity and specificity, LDH is only used to distinguish acute from subacute MI in patients with positive troponins while CK and CK-MB are negative (51). Moreover, a LDH-1:LDH-2 ratio >1 is reported to be specific for diagnosing AMI (51).

#### Creatine Kinase

Creatine kinase (CK) activity was considered a better predictor of myocardial injury and an independent indicator of AMI for 20 years (8). CK is a dimeric enzyme, consisting of two subunits, M and B, and has three isoenzymes, CK-BB (CK1), CK-MB (CK2), and CK-MM (CK3) (52). Among them, only CK-MB is found in the heart, but CK-MB is also detected in other organs, such as uterus, tongue, etc. (84). When released into the blood, CK-MB can be divided into two groups, MB1 and MB2. When AMI occurs, MB2 passes into blood companying with significant change in the MB2:MB1 ratio. An MB2:MB1 ratio ≥1.5 is considered as an indicator of AMI (5). CK-MB is an excellent biomarker in diagnosis of AMI during the first 6 h, and at the same time, the negative predictive value during the first 6 h is 97% (5). Furthermore, it was reported that the CK-MB relative index (CK-MB/total CK × 100) could be used for diagnosis of MI. If this index is 2.5% or above, CK-MB has a great possibility released from heart (53). Finally, the total CK and CK-MB levels are correlated with infarct size and provide possibility to predict prognosis. CK-MB, however, cannot detect minor myocardial damage (54).

#### Myoglobin

Myoglobin is a biomarker for early detection and/or exclusion of cardiac injury because the serum level of myoglobin rises in the first 30 min after the onset of an acute event (9). Negative values are more meaningful in the clinic than positive values due to its low-specificity (55).

#### Cardiac Troponin

Increased serum cardiac troponin (cTn) level is the gold standard for AMI diagnosis. Combined changes of cTn with clinical manifestations and ECG could initially identify AMI in the early stage after the onset of chest pain, which can decrease mortality significantly.

Cardiac troponin I (cTnI) is presented in cardiac muscle tissue. Cardiac troponin T (cTnT) is expressed in both skeletal and cardiac myocytes. There is no report that cTnI increases after non-cardiac tissues are damaged while cTnT is more complicated because elevated cTnT may be derived from skeletal muscles. Thus, cTnI is more specific in diagnosing MI (2, 85, 86). High sensitive (hs)-cTn assays measure cTn concentrations 5- to 100-fold lower than conventional assays. Anda et al. suggested that use of risk stratification thresholds for hs-cTnl could identify patients with suspected acute coronary syndrome and at least 2 h of symptoms as low risk at presentation irrespective of age and sex (87). Due to higher sensitivity and specificity compared with others biomarkers, cTnI plays an important role in diagnosis of MI and high-sensitivity (hs)-cTn assays are routinely used in clinic (46).

#### Heart Type Fatty Acid-Binding Protein

Fatty acid-binding proteins (FABPs) belong to a family of proteins that are responsible for the transportation of fatty acids and lipophilic materials into or out of cells (88). There are several types of tissue-specific FABPs, including heart-type, liver-type, intestinal-type, epidermal-type, brain-type, ileal-type, myelin-type, adipocyte-type, and testis-type FABPs (88). Heart type fatty acid-binding protein (H-FABP) is a small (15 kDa) soluble protein. Despite not being cardiac-specific, H-FABP plays an essential part in metabolism of fatty acid (FA) inside cardiomyocytes and present at high concentrations in cardiomyocytes cytoplasm (89). Other tissues such as skeletal muscle, brain, and kidney also produce it, although at a lower concentration than in myocardium (90). Watanabe indicated that a “decreased immunoreactivity for H-FABP may be a good histological biomarker of damaged cardiomyocytes” (22). Serum concentration of H-FABP increases in the first 1–2 h after symptom onset, which provides possibility for early diagnosis of AMI, and peak concentration is achieved in approximately 5–10 h, after which it returns to its reference range within 24–36 h (91). Notably, H-FABP demonstrates different sensitivity and specificity at different cutoff, and the highest sensitivity of H-FABP is measured at 4 h (88%) (56). However, its sensitivity and specificity are lower than hs-cTn and thereby it cannot be used as a standalone biomarker for AMI diagnosis. Further studies may help to determine the most precise cutoff point of H-FABP for AMI diagnosis and its additional usage in cardiovascular emergencies (57).

Recently, more attention has been paid on H-FABP as a biomarker for immediate myocardial injury and even for relatively long-term post-ischemic prognosis. Although H-FABP has relatively poor effects on AMI diagnosis, there is evidence that H-FABP plays an important role in evaluating in-stent restenosis and achievement of heart reperfusion after ischemic attack (57, 58, 92). Huang et al. (59) also found H-FABP to be a more sensitive biomarker than cTnI and CK-MB for sensing post-ischemic myocardial reperfusion injury. Additional analysis showed that H-FABP could be a biomarker that can independently predict adverse cardiac events on different levels and provide information for a risk evaluation for clinicians (60, 93, 94). Furthermore, a high negative predictive value of H-FABP test can help to rule out AMI earlier (61), which can reduce hospitalizations and expenses. It is worth mentioning that in 2018, Jo et al. proved that H-FABP would be a more useful biomarker to detect myocardial ischemic injury than CK-MB and cTnT (95). Based on the properties of H-FABP, the combination of H-FABP with other biomarkers, for instance, H-FABP at the early stage and cTnI at the late stage of AMI, may achieve better diagnostic and prognostic significance.

#### Myosin-Binding Protein C

The myosin-binding protein C family consists of three isoforms (96). Notably, the cardiac myosin-binding protein C (cMyC) is expressed in the heart specifically (24, 25). cMyC is more abundant in myocardial tissue and in blood circulation than cTn. cMyC is essential in assembly and function of cardiac sarcomere (26, 27, 63). Due to the delayed appearance of cTn, patients who demonstrate acute chest pain need to test repeatedly to determine AMI while cMyC appears earlier and rises faster in AMI patients (97). Thus, cMyC has an advantage over cTn for early diagnosis of AMI.

It is reported that cMyC rises and falls more rapidly after AMI in patients with vascular risk factors and/or underlying chronic heart disease (98). The ability to distinguish patients suffering MI or acute chest pain by cMyC is similar to that of hs-cTnT and hs-cTnI and superior to s-cTnI (99). In patients presenting <3 h of chest pain onset, cMyC is superior to hs-cTnT (99). In short, cMyC is a promising AMI biomarker with the strengths of ruling in/out AMI more effectively at early onset.

### Biomarkers Induced by MI Incidence

#### Interleukins

Interleukins (ILs) are a group of cytokines that are expressed by leukocytes. ILs can be divided into four major groups based on distinguishing structural features. The human genome encodes more than 50 interleukins and related proteins.

IL-1 family is a group of 11 cytokines, which induces a complex network of proinflammatory cytokines *via* expression of integrins on leukocytes and endothelial cells, and regulates and initiates inflammatory responses. In 2004, Patti et al. evaluated that interleukin-1 receptor antagonist (IL-1Ra) increased early in patients with AMI, especially in those with premonitory infarction and symptom onset ≤3 h, and preceded other biomarkers of necrosis (15). IL-1Ra may be an important early adjuvant toward diagnosis of AMI in the emergency department.

IL-32 is a newly discovered inflammatory cytokine with eight isoforms in most mammals. IL-32 is found to be highly expressed in human atherosclerotic plaques and significantly increased in patients with heart failure after MI (16, 17). Furthermore, the soluble IL-1 receptor 2 (sIL-1R2), IL-1, IL-6 plays an important role in myocardial remodeling after MI (64, 65, 100). For the time being, the IL-related therapy of MI is effective, indicating that further research on ILs is needed for MI diagnosis and therapy.

#### Insulin-Like Growth Factor 1

Insulin-like growth factor 1 (IGF-1) is an anabolic hormone that controls growth and metabolism of many cell types. In 1997, Scheinowitz indicated that IGF may protect cardiac function after AMI (23). The majority of circulating IGF-1 molecules combine with IGF binding proteins (IGFBPs), which can modulate IGF-1 binding to the IGF-1 receptor (IGF1R) (101). Evidence shows that IGF-1 levels are correlated with occurrence of coronary heart disease, which functions possibly by affecting atherosclerosis progression (102).

Free IGF-1 is inferior to CK-MB as an indicator of myocardial damage (103). However, IGF-1 plays an important role in some other aspects of MI. IGF-1 can affect vascular function and atherosclerosis by anti-inflammatory and anti-apoptotic actions as well as by stimulating angiogenesis (67–69, 104). At the same time, there is also evidence indicating that IGF-1 has indirect effects on the cardiovascular system by increasing insulin sensitivity (69–71). On the one hand, IGF-1 prevents recruitment of monocytes/macrophages from atherosclerotic plaques, production of proinflammatory cytokines, conversion of macrophages into lipid-laden foam cells, and extracellular matrix degradation. IGF-1 also promotes smooth muscle cell (SMC) migration, proliferation, and SMC-dependent matrix deposition, all of which may contribute to IGF-1-induced reduction in plaque burden and increase in plaque stability (72). By the mechanism described above, IGF-1 stabilizes plaque so as to decrease MI events. Furthermore, IGF-1 treatment is effective to reduce adverse cardiac remodeling after cardiac ischemia/reperfusion injury, when IGF-1 is administered systemically (105). Finally, Yao et al. found that combination of hepatocyte growth factor (HGF) and IGF-1 promote connexin 43 expression and improve ventricular arrhythmia after MI in a rat model (106), which may exhibit therapeutic potential for ventricular arrhythmias after MI.

#### Vascular Endothelial Growth Factor

Vascular endothelial growth factor (VEGF) is a highly specific growth factor for vascular endothelial cells, which can promote vascular permeability, extracellular matrix denaturation, vascular endothelial cell migration, proliferation, and angiogenesis. VEGF family include VEGF-A, VEGF-B, VEGF-C, VEGF-D, VEGF-E, and placenta growth factor (PGF).

VEGF-A, which is actively produced in damaged myocardium to foster angiogenesis and tissue repair, is a major effector of endothelial junction disruption and vascular leakage (42, 107, 108). Clinical studies on VEGF-A in patients with AMI have demonstrated that low levels of circulating VEGF-A are an independent risk factor for adverse clinical outcomes after AMI (43, 44). VEGF-A 165b is the main anti-angiogenic isoform of VEGF-A and associates with infarct size in patients with AMI and dysregulated VEGF-A 165b in aging endothelial cells contribute to the risk of coronary heart disease (109–111). Study shows that the assessment of VEGF-A 165b combined with VEGF-A may predict main adverse cardiovascular events (MACEs) in clinical practice (112) and VEGF-A 165b might play a negative regulatory role in AMI as an inhibitor of angiogenesis in myocardium. According to that, therapy aimed at VEGF-A 165b might be significant to reperfusion of myocardium after STEMI (109). In addition, a low VEGF-C value may independently predict all-cause mortality in patients with suspected or known CHD (113).

In summary, VEGF has little effect on diagnosis of MI while it has significant effect on prognosis and MI treatment.

#### Matrix Metalloproteinases

Matrix metalloproteinases (MMPs) are a group of zinc ion-dependent proteases that degrade collagen and proteoglycans and play an important role in the development of atherosclerosis (114). MMPs play a pivotal role in post-myocardial infarction cardiac remodeling as well as in the development of adverse outcomes (115). Circulating MMP-28, a new member in the family of MMPs, could be considered a predictor for short-term prognosis in patients with myocardial infarction (73).

### Biomarkers Preexisted Before MI Occurred

#### Glucose

A recent study reported that in a well-treated contemporary population of AMI patients, 42% of patients without diabetes have elevated admission plasma glucose levels and AMI event rates are increased with the elevated admission plasma glucose levels (116). In addition, fasting blood glucose levels are significantly increased in patients suffering AMI (117). These findings highlight the importance to research and develop the circulating biomarkers usable for MI prevention.

#### Aspartate Aminotransferase

Aspartate Aminotransferase (AST) catalyzes the reversible transfer of an α-amino group between aspartate and glutamate. AST plays an important role in amino acid metabolism. AST is found in the liver, heart, skeletal muscle, kidneys, brain, and red blood cells. In 1954, AST is established as a biomarker in assisting AMI diagnosis (5). However, it was abandoned because increased serum AST levels are associated with various diseases and also present in both pre- and after AMI events.

### RNAs

#### microRNAs

A whole new understanding of miRNA began in 2003 when Ambros showed that miRNA participates in regulating development process in worms (118). MicroRNAs (miRNAs/miRs) are a class of tissue-specific or cell-specific small (19–25 nucleotides) non-coding RNAs, which have effects on various biological processes including cell growth, proliferation, differentiation, and apoptosis (119, 120). MicroRNAs are circulating in plasma/serum and have been tested as biomarkers for cardiovascular diseases (30). Among them, microRNA-499 has been shown to be expressed in myocardium and skeletal muscle in mammals, and the blood samples from patients have high levels of microRNA-499 before the AMI events (31, 32). Circulating miR-499 has good sensitivity and specificity for differentiating AMI from non-AMI (0.84 and 0.97, respectively) and it is considered for early diagnosis of AMI (33).

By detecting plasma concentration of microRNA-145 in patients, Zhang et al. indicated that low microRNA-145 levels correlate inversely with the severity of AMI (34). They also speculated that circulating microRNA-145 might not only be of use in diagnosing MI but could also potentially be helpful in prognosticating cardiac function and the risk of developing heart failure. In 2015, Jia et al. indicated that miR-125b-5p and miR-30d-5p have a value for early diagnosis of AMI, and miR-30d-5p might37 have a higher diagnostic value than cTnI (35). MicroRNA-208b (34), microRNA-133a (121), miR-486 as well as miR-150 (36), and microRNA-21 (37) may be novel biomarkers used for the diagnosis of AMI.

#### Long Non-coding RNAs

Zhong et al. indicated that differential expression of long non-coding RNAs (LncRNAs) would be helpful to understand molecular mechanism of AMI and might be useful biomarkers for non-invasive diagnostic application (122). LncRNAs are a set of RNA transcripts containing more than 200 nucleotides, which cannot transcript protein but have same effects with miRNAs (123). It has been reported that lncRNAs are involved in regulation of cardiac development, pathogenesis of heart failure, and the role of cardiovascular aging (38, 39). Previous studies demonstrated that the serum levels of three lncRNAs, namely H19, MIAT, and MALAT1, are significantly increased in AMI patients when compared with healthy volunteers, which indicates that these lncRNAs are promising biomarkers for the diagnosis of AMI (124).

#### S100 Protein

S100 belongs to the family of EF-hand proteins and is a calcium-binding protein with a low molecular weight of 10–12 kD. Its amino acid sequence is highly conserved in vertebrates and it has high homology with calmodulin and other EF-hand type calcium-binding proteins. At present, there are at least 21 different types of S100 proteins. They are also named as damage-associated molecular pattern molecules (DAMPs). S100 protein consists of two isomeric subunits(α/β) with αα, αβ, and ββ combinations (125).

S100 proteins are normally present in cells derived from the neural crest, chondrocytes, adipocytes, myoepithelial cells, macrophages, Langerhans cells, dendritic cells, and keratinocytes. It has been shown that heart and skeletal muscle are rich in S100A while most S100B is found in the brain (10, 126). S100 proteins have been implicated in a variety of intracellular and extracellular functions. S100 proteins are involved in regulation of protein phosphorylation, transcription factors, Ca^2+^ homeostasis, the dynamics of cytoskeleton constituents, enzyme activities, cell growth and differentiation, and the inflammatory response.

Several members of the S100 protein family are useful as biomarkers for certain cancers (127). Further, S100 proteins are biomarkers for inflammatory diseases and can mediate inflammation and act as antimicrobials (128). Serum S100A0 is a useful biomarker for diagnosing AMI, which is also better than CK-MB for differentiating AMI from angina pectoris (11). S100A1 is abundant in the heart, especially ventricular cardiomyocytes (12), which can be used to detect the postmortem diagnosis of AMI at an early stage (13). S100A4 expression protects cardiac myocytes against myocardial ischemia and is required for stabilization of cardiac function after MI (14). In 2011, serum levels of S100B, S100A6, S100P, and soluble receptor for advanced glycation endproduct (sRAGE) were analyzed in 882 patients. It was found that serum levels of S100B, S100A6, and S100P are associated with ischemic myocardial injury in acute coronary syndrome (ACS), and expression of these S100 proteins is related to myocardial infarct size (129). As for S100B, it plays an important role in down-regulating cardiac myocyte hypertrophy and is an attractive therapeutic target for treatment of cardiovascular disease (74). In addition, S100B expression may affect cardiac metabolism in diabetic post-MI remodeling and function (75).

More attention should be directed toward S100 as early diagnostic biomarker for MI prevention.

#### Heparanase

Heparanase (HPA) is an endo-b-D-glucuronidase capable of degrading heparan sulfate (HS) and heparin side chains (130), whose expression is particularly in placenta, platelet, keratinocyte, and active cells of the immune system. HPA plays a role in tumor growth, angiogenesis, cell invasion, and activation of the coagulation system (131, 132). Here we mainly discuss its effects on atherosclerosis, stenosis, and thrombosis, which are all associated with arterial plaque development and rupture (133).

Blich et al. found that HPA levels increase nearly 9-fold in patients with AMI while 3-fold in patients with stable angina (SA) compared to healthy individuals (47). They found that high levels of HPA promotes plaque toward vulnerability, which is pathological basis for MI. Furthermore, HPA functions as a mediator to enhance expression of tissue factor and generation of factor Xa, which are two critical components in blood coagulation (77, 134). Thrombosis caused by rapid blood clotting might lead to disruption of coronary blood blow thereby onset of AMI. In addition, HPA is a predictive biomarker for high thrombus burden in patients with STMI (135). HPA levels are associated with plaque vulnerability and progression and may thus be considered as a pre-diagnostic biomarker and potentially therapeutic target for prevention of acute heart diseases.

#### PIK3C2A

PIK3C2A belongs to phosphoinositide 3-kinases (PI3Ks), which is a family of enzymes that phosphorylate the 3′-OH position of the inositol ring of phosphatidylinositol (PI), and regulate a broad range of signaling pathways (136). A retrospective study showed that the level of PIK3C2A gene expression in patients with AMI is significantly lower than that of healthy individuals. Low expression of PIK3C2A is an independent risk factor and could serve as a potential biomarker to predict the risk of AMI (78).

#### Copeptin

Copeptin, a neuropeptide, has attracted interest for its use as part of a dual marker strategy in combination with cTn for the early rule-out of chest pain patients with suspected NSTEMI (137). A large pooled cohort showed that copeptin below cut-off in combination with hs-cTn below the upper limit of normal range may be used in more than 2.4-times more patients presenting with suspected acute coronary syndrome than a single biomarker strategy based on very low hs-cTn (79). Furthermore, men with suspected NSTEMI have higher copeptin levels, and certain predictors of copeptin elevation are gender-specific (80).

#### Mitsugumin 53

Mitsugumin 53 (MG53), a muscle-specific protein belonging to the tripartite motif family, has been demonstrated to protect the heart against oxidative injury. A study demonstrated that elevated serum MG53 levels have a significant adverse outcome after a 3-year follow-up among patients with STEMI. The measurement of MG53 could be used as a novel biomarker to improve the current means of risk stratification of AMI (81).

#### The Urine Albumin-To-Creatinine Ratio

The urine albumin-to-creatinine ratio (uACR) has been verified to be independently associated with increased long-term risks of cardiovascular and total mortality in survivors of MI (28). The serum albumin-to-creatinine ratio (sACR) was found to be an independent prognostic biomarker in patients with AMI on admission to the emergency department and a useful biomarker for early risk stratification of patients with AMI (82).

#### The Other Functions of MI Biomarkers

Except for MI diagnosis, the biomarkers mentioned above are also involved in atherosclerosis progression, evaluation of cardiac function, prognosis, etc. (Table 5).

**Table 5:** Other biological functions of MI circulating biomarkers.

**Biomarkers**		**Application value**	**References**
S100	S100A8	Prevents myocardial ischemia and stabilize cardiac function after MI	(14)
	S100B, S100A6, S100P	^*^Associate with ischemic myocardial injury in ACS ^*^ Relate to myocardial infarct size	(129)
	S100B	^*^Down-regulate cardiac myocyte hypertrophy and apoptosis ^*^Affect myocardial metabolism	(74, 75)
IL	sIL-1R2, IL-1, IL-6	Associated with myocardial remodeling after MI	(64, 65, 100)
H-FABP		^*^Involved in in-stent restenosis ^*^Evaluate the degree of heart reperfusion after ischemic attack ^*^Predict adverse cardiac events and provide risk evaluation	(57, 58, 60, 92–94)
IGF-1		^*^Affect atherosclerosis progression ^*^ Reduce adverse cardiac remodeling after cardiac I/R injury ^*^ Improve ventricular arrhythmia after myocardial infarction combined with HGF	(102, 105, 106)
microRNAs	microRNA-145	Prognosticate cardiac function and the risk of developing HF	(40)
	microRNA-1,microRNA-133, microRNA-208	Diagnose sudden death due to early AMI sensitively	(138)
	microRNA-124	Reduce cardiomyocyte apoptosis following MI	(41)
	microRNA-208b	Reduces post-infarction myocardial fibrosis in	(139)
	microRNA−93	Inhibit cardiac remodeling and HF	(140)
	miR-24-3p	Exert cardioprotective effects in myocardial I/R injury	(141)
VEGF	VEGF-A	Inversely correlated with LVEF after MI at 6-month follow-up	(45)
	VEGF-C	Predict all-cause mortality independently	(113)

Many circulatory factors are associated with function and prognosis in patients with cardiovascular diseases (142, 143). Many other functions for potential serum MI biomarkers have been reported. For example, the levels of protooncogene tyrosine-protein kinase (SRC), C-C motif chemokine ligand 17 (CCL17), and chymotrypsin C (CTRC) are all significantly decreased in extracellular vesicles (EVs) lysates from MI patients but remain unaltered in the normal control plasma samples (144). The EVs isolated from plasmas provide additional diagnostic value and improve pathophysiological understanding compared to plasma alone in the context of MI, but further study on EVs is required. Moreover, by using proteomic analysis, elevated serum levels of Pregnancy Zone Protein (PZP) and Leucine-Rich Alpha-2-Glycoprotein (LRG) are shown to be independent risk factors for early-onset MI. Therefore, inflammation-associated LRG and PZP may be novel MI biomarkers (145). Furthermore, there is evidence indicating that galectin-3 may be a promising biomarker for evaluation of severity and prognosis of AMI (146). Recently, it was demonstrated that the levels of microparticles (MPs), especially CD31^+^CD42^−^EMPs and CD144^+^EMPs, have the order of normal subjects < stable angina < unstable angina < MI, indicating that MPs have the potential capacity to distinguish stable angina, unstable angina, and MI (147).

## Conclusions and Outlook

MI is an aging-related systems disease caused by multiple genetic and environmental factors in addition to lifestyles (148, 149). It is understandable that high-sensitivity cardiac troponin (hs-cTn) is the current golden standard for AMI diagnosis since it is the specific heart tissue damage product. However, other circulating biomarkers are needed for prevention, prognosis, and treatment effect monitoring purposes.

Both glucose and HPA have pre-diagnostic properties. It has been proved that fasting blood glucose is an independent risk factor for Gensini score in AMI patients (150). In addition, a cohort study has shown that MI with diabetes mellitus tend to develop cardiogenic shock and have worse outcomes (151). A 10-year cohort study demonstrates that relatively high but clinically normal serum glycated hemoglobin A1c (HbA1c) and thyrotropin (TSH) may increase risk of coronary heart disease (CHD) (152). Most cancer biomarkers, such as CA199 and carcinoembryonic antigen (CEA), are either specific glycan structures or heavily glycosylated proteins (153). Among them, elevated carbohydrate antigen 125 (CA125) can be used to predict mortality risk at 6 months following AMI (154), which is consistent with our previous findings that the circulating CA125 levels are higher in fibrosis-associated diseases than in most types of cancers (155).

We classified the MI biomarkers for their diagnostic, prognostic, and preventive purposes and also discussed other biological functions of the biomarkers in current review. However, certain biomarkers belong to more than one category listed in Tables 1–5. Since the development of MI is a long process accompanied with multiple changes of the systems, biomarkers preexisting in blood circulation before MI incidents should be emphasized in research and development for MI prevention in the near future.

## Author Contributions

YW drafted the manuscript and drew the figures. NP, YA, MX, LT, and LZ revised the manuscript for important intellectual content. YW and LZ were responsible for manuscript concept and design and edited the final manuscript. All authors approved the final version of the manuscript.

## Conflict of Interest

The authors declare that the research was conducted in the absence of any commercial or financial relationships that could be construed as a potential conflict of interest.
